# Dr. J. N. Johnson

**Published:** 1823-12-01

**Authors:** 


Lately deceased at his house in the Albany, Dr. J. N. Johnson, Fellow of the Royal College of Physicians.

